# Manifestations of Myelinating Oligodendrocyte Glycoprotein Antibody-Associated Disease: A Rare Case of Suppurative Meningoencephalitis

**DOI:** 10.7759/cureus.56941

**Published:** 2024-03-26

**Authors:** Huiyao Xiang

**Affiliations:** 1 Department of Neurology, The First College of Clinical Medical Science, China Three Gorges University, Yichang, CHN

**Keywords:** central nervous system, immunoglobulin, inflammatory and demyelinating disease, suppurative meningoencephalitis, myelin-oligodendrocyte glycoprotein (mog)

## Abstract

Myelinating oligodendrocyte glycoprotein antibody-associated disease (MOGAD) is an inflammatory demyelinating disease of the central nervous system (CNS) mediated by MOG antibodies (MOG-IgG). It is associated with autoimmunity and encompasses various syndromes. However, manifestations presenting with symptoms of suppurative meningoencephalitis are rare. In this case, we admitted an 80-year-old male patient who presented with fever, headache, mental and behavioral abnormalities, and disturbance of consciousness. The cerebrospinal fluid (CSF) examination revealed elevated levels of leukocytes and protein, while magnetic resonance imaging (MRI) of the brain showed an abnormal signal in the parietal lobe surrounding the posterior horn of the right lateral ventricle. The patient tested positive for serum MOG-IgG, confirming the diagnosis of MOG-IgG-related meningoencephalitis. The treatment included intravenous immunoglobulin, glucocorticoids, third-generation cephalosporins, and immunosuppressants. Following the treatment, the patient experienced complete recovery.

## Introduction

Myelin oligodendrocyte glycoprotein (MOG), a member of the immunoglobulin superfamily, is a myelin protein expressed exclusively on the outer surface of myelin sheaths and oligodendrocyte membranes in the central nervous system (CNS) [1]. MOGAD is an autoimmune disease that has been proposed in recent years to manifest as CNS demyelination in both adults and children independent of multiple sclerosis and neuromyelitis optica spectrum disorders [2]. MOGAD may be preceded by a predisposing factor such as infection, which accounts for 37%-70% of cases [3], and most commonly viral infections, including influenza virus, Epstein-Barr virus, herpes simplex virus, acute respiratory syndrome coronavirus, and novel coronaviruses [4-6]. In contrast, positive expression of MOG-IgG induced by suppurative meningoencephalitis is relatively rare. We report a case of suppurative meningoencephalitis with MOG-IgG positivity to increase clinicians' awareness of MOGAD as a clinical phenotype for early diagnosis and treatment.

## Case presentation

An 80-year-old male patient was admitted to the hospital with a fever for 4 days. His temperature reached up to 39.5℃ and was accompanied by headache, gradual speech and confusion, and visual and auditory hallucinations. There was no slurred speech, dysphagia, choking on liquid, blurred vision, double vision, unilateral limb weakness, hypesthesia, loss of consciousness, limb convulsions, staring eyes, foaming at the mouth, and urinary and fecal incontinence; his Glasgow Coma Score (GCS) was 14 (4+4+6). The patient had a history of hypertension and no other specific medical history.

Physical examination showed a temperature of 39.0°C. Other internal systems showed no abnormalities. A neurological examination showed that the patient was coherent and had clear speech but poor concentration. The patient also displayed poor temporal and spatial orientation, verbosity, disorganized speech, and visual and auditory hallucinations. In addition, the patient presented with nuchal rigidity and a positive Kernig's sign. All other neurological examinations were normal.

**Figure 1:**
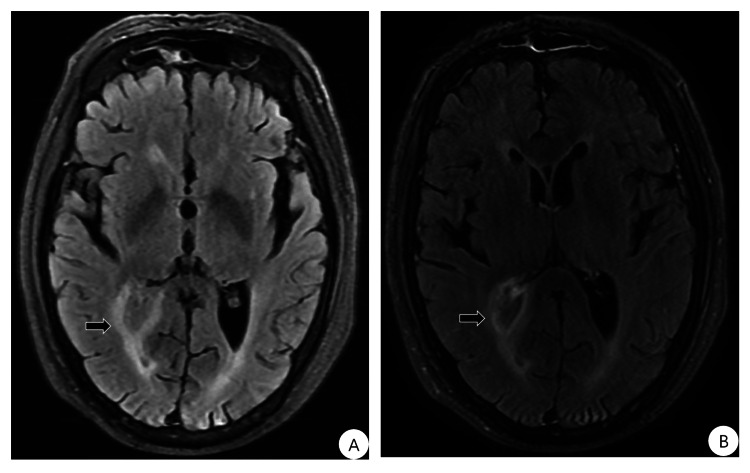
Brain MRI showed increased signal around the posterior horn of the right lateral ventricle in the right parietal lobe in both T2/FLAIR and contrast-enhanced images. T2/FLAIR (A) and enhanced FLAIR (B).

Laboratory examination showed normal *Mycobacterium tuberculosis* DNA and T-cell tests, respiratory virus nucleic acid tests, blood cultures, thyroid function, tumor markers, autoantibody profile, antibodies to human immunodeficiency virus antigens, *Syphilis* antibodies, antibodies to immunoglobulins, computed tomography scan of the entire abdomen, and electroencephalogram. CSF examination showed a pressure of 280 mmH_2_O, a pale yellow slightly cloudy appearance, and a leukocyte count of 1,215×10^6^/L (normal range is 0-8 ×10^6^/L) with 62.0% of multinucleated cells. CSF cytology showed inflammation with predominant lymphocytes, while the protein content was 203.9 mg/dl (normal range 15-45 mg/dl), glucose was 1.49 mmol/L (normal range 2.2-3.9 mmol/L), and chloride was 118.9 mmol/L (normal range 118-132 mmol/L). CSF acid-fast stain, ink stain, cryptococcal capsular antigen detection, culture, and virus antibody (for example Japanese encephalitis virus, cytomegalovirus, EB virus, influenza virus, parainfluenza virus, herpes zoster virus, herpes simplex virus, adenovirus, rubella virus) were normal. Plain and enhanced brain MRI scans showed abnormal signals in the parietal lobe surrounding the posterior horn of the right lateral ventricle (Figure 1). The MRI scans of the optic nerve, cerebrovascular system, and cervical thoracic spine revealed no evidence of abnormal signals. Therefore, we diagnosed suppurative meningoencephalitis and treated it with cefotaxime sodium 2.0g every 12 hours and methylprednisolone 20mg/day. After the treatment, the body temperature gradually decreased, and the headaches and neuropsychiatric symptoms gradually improved.

**Figure 2:**
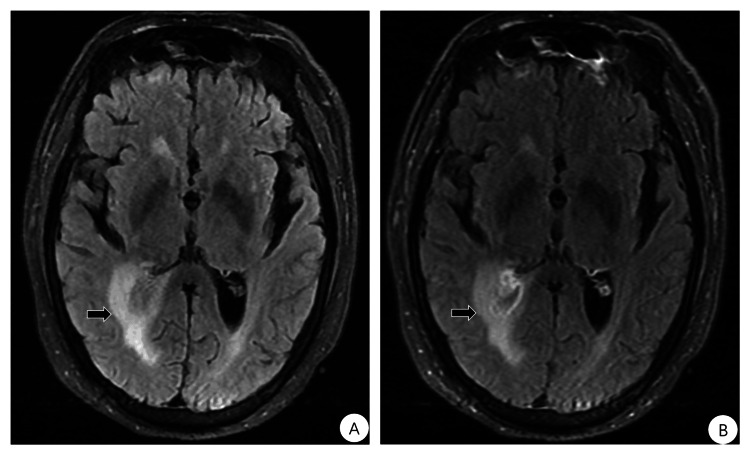
Follow-up brain MRI after one week of treatment with cefotaxime 2g every 12 hours and methylprednisolone 20mg/day indicated a slight enlargement of the lesion. T2/FLAIR (A) and enhanced FLAIR (B).

One week later, the patient's condition worsened with altered consciousness, lethargy, and gradual loss of consciousness. His GCS score was 9 (2+3+4), and a repeat CSF examination showed a pressure of 200 mmH _2_ O, a leukocyte count of 660×10 ^6^ /L, protein content of 102.3 mg/dl, a glucose content of 3.78 mmol/L, and a chloride content of 126.8mol/L. MOG antibody was positive in serum (titer: 1:32, cell-based assay method), but negative in CSF. Autoimmune encephalitis antibody, paraneoplastic syndrome-associated antibody, aquaporin-4 antibody, and oligoclonal bands in CSF and blood were normal. Re-examination of the plain and enhanced brain MRI scans showed that the abnormal signal in the parietal lobe surrounding the posterior horn of the right lateral ventricle was slightly increased compared with the previous images (Figure 2). Based on the above results, we finally diagnosed MOGAD. The patient was treated with immunoglobulin 0.4 g (kg/d) for 5 days. On the third day of immunoglobulin treatment, the patient regained consciousness, and the GCS score increased from 9 to 12. Subsequently, prednisone 20mg was taken in the morning, and mycophenolate mofetil 0.25g was taken orally twice a day. During hospitalization, the patient also received intracranial pressure reduction, gastric protection, potassium and calcium supplementation, deep vein thrombosis prophylaxis, and nutritional support. When the patient was discharged two weeks later, there were no positive findings on neurological examination. He was discharged from the hospital with regular follow-up visits, the last of which was nearly 2.5 months later, with no recurrence and a negative repeat test for serum anti-MOG antibodies.

## Discussion

The patient in this case study was an 80-year-old man with an acute onset of illness. Major symptoms included high fever, headache, speech and logic disorder, and auditory and visual hallucinations. Neurological examination revealed nuchal rigidity and a positive Kernig's sign. The initial lumbar puncture revealed high CSF pressure with a leukocyte count greater than 1000×10^6^/L and an elevated protein level. Brain MRI showed abnormal signals in the posterior horn of the right lateral ventricle. Based on these findings, a preliminary diagnosis of suppurative meningoencephalitis was made. Previous studies have shown that *Streptococcus pneumoniae* is the most common cause of meningitis in elderly patients and is associated with adverse disease outcomes [7]. However, a recent prospective national cohort study in the Netherlands showed that *Listeria monocytogenes*, *Staphylococcus aureus*, and *Escherichia coli* were more common in patients over 80 years of age [8]. While China is an area with a high incidence of bacterial meningitis with an incidence rate of 20-80 cases per 100,000 population [9], there are no national guidelines or expert consensus on the diagnosis and treatment of bacterial meningitis. Our treatment regimen, based on guidelines from the United States and large clinical trials from Sweden and the Netherlands [10], uses a third-generation cephalosporin (cefotaxime) in combination with a low-dose hormone (methylprednisolone).

However, during treatment, the patient's level of consciousness changed, and a repeat lumbar puncture showed a decreasing trend in CSF pressure, cell count, and protein content, with worsened clinical symptoms. Re-examination of the brain MRI showed a slight enlargement of the lesion. We considered possible comorbidity with an immune-related disease and performed relevant antibody tests, which showed positive MOG antibodies in the serum. The final diagnosis of MOGAD.

The differential diagnosis must include other pathogens that cause meningoencephalitis, such as viruses (Japanese encephalitis virus, cytomegalovirus, Epstein-Barr virus, Influenza virus, Parainfluenza virus, herpes zoster virus, herpes simplex virus, Adenovirus, and Rubella virus), *Mycobacterium tuberculosis*, and *Cryptococcus neoformans*. The patient had an acute onset of illness with high fever, headache, abnormal mental behavior and level of consciousness, and a CSF leukocyte count greater than 1000×10^6^/L. In addition, no other causative organisms were identified by pathogenetic examination, including blood and CSF, which does not support the diagnosis of meningoencephalitis caused by other pathogenic organisms. The second is autoimmune encephalitis. The disease usually has a subacute onset and is often accompanied by prodromal symptoms such as fever and headache. Clinical symptoms are variable and include mental and behavioral abnormalities, cognitive impairment, memory loss for recent events, seizures, decreased level of consciousness, and coma. In addition, an MRI of the brain showing abnormal signals in the limbic system and positive anti-neuronal cell antibodies can confirm the diagnosis. However, the combination of our relevant ancillary findings did not support the diagnosis of the disease.

MOGAD is a recently discovered autoimmune demyelinating disease of the central nervous system. The most common onset features are optic neuritis, acute disseminated encephalomyelitis, and transverse myelitis [11]. Less common presentations include cerebral cortical encephalitis (often with seizures), brainstem and cerebellar demyelinating attacks, tumefactive brain lesions, cerebral mono-focal and polyfocal CNS deficits associated with demyelinating lesions, cranial neuropathies, and progressive white matter damage [12]. Of these, acute disseminated encephalomyelitis is more common in children, whereas optic neuritis and isolated myelitis are more common in adults, and adults are more likely to relapse [13].

Previous literature has reported that MOGAD is often preceded by a history of viral infection or vaccination, while bacterial infections are relatively rare [4,5]. The association between viral or bacterial infections and MOGAD is unknown. Infection-associated cytokine and chemokine markers are important components of MOGAD [14]. In the inflammatory microenvironment of the peripheral blood, MOG-specific T cells, especially CD4+ cells, can be activated by bypass activation systems or molecular mimicry mechanisms [15]. Activated T cells further lead to the opening of the blood-brain barrier, allowing autoantibodies and immune cells to enter the CNS and cause demyelinating lesions [3]. It has also been shown that post-infectious cytotoxicity can lead to oligodendrocyte demyelination through a mechanism that alters membrane potential and inhibits potassium channels [16].

Serum MOG-IgG is an important marker for the diagnosis of MOGAD with high specificity [17]. MOG-IgG titers correlate with positive predictive value, specificity, disease severity, and recurrence rate [12]. Patients with high or persistently positive serum MOG-IgG titers are more likely to relapse [12]. However, if the antibody titer is less than 1:20 and the clinical symptoms are atypical, the diagnosis of MOGAD should be made with caution [17]. Nevertheless, low positive MOG-IgG is reported to be meaningful in the correct clinical context such as in patients with encephalitis. Additionally, patients with acute attacks of unilateral cortical encephalitis are considered to have a high pre-test probability for MOGAD and a low risk of false-positive MOG-IgG results [17]. In this case, the patient's MOG-IgG titer of 1:32, the typical clinical picture of encephalitis syndrome, and the exclusion of other suspected diseases allowed a more reliable diagnosis.

Treatment of MOGAD generally follows the therapeutic process for CNS demyelination. In the acute phase, the first choice is high-dose glucocorticoid shocks, which are gradually tapered and maintained at low doses. Next, treatments such as intravenous immunoglobulin or plasma exchange may be considered. However, a multicenter study showed that patients who received preferred immunoglobulin therapy had lower relapse rates [18]. In remission, commonly used maintenance therapies include oral steroids, immunosuppressive agents such as azathioprine, mycophenolate mofetil, and B-cell-targeting biologics (such as rituximab and tocilizumab) [4]. The decision to administer immunosuppressive therapy depends on the patient's response to the initial treatment, the severity of the initial symptoms, the risk of short-term disability (associated with the initial attack or accumulation of attacks), the risk of long-term immunization, and age [2]. In the present case, because of the patient's advanced age and the severity and rapid progression of initial symptoms, we chose immunoglobulin shock therapy, followed by a regimen of low-dose hormones in combination with oral mycophenolate mofetil. In the subsequent follow-up, the patient had no relapse and no adverse effects, with a favorable prognosis.

## Conclusions

In conclusion, this article reports a patient with suppurative meningoencephalitis who tested positive for MOG-IgG. In patients presenting with symptoms of first-episode suppurative meningoencephalitis, if symptoms do not improve or continue to worsen after standard anti-infective therapy, it is necessary to perform anti-MOG antibody testing, and adequate pathogenetic examination and differential diagnosis to provide effective treatment as early as possible, thereby improving prognosis.
